# Study of Cardiovascular Outcomes in Renal Transplantation: A Prospective, Multicenter Study to Determine the Incidence of Cardiovascular Events in Renal Transplant Recipients in Ontario, Canada

**DOI:** 10.1177/2054358117713729

**Published:** 2017-06-14

**Authors:** Christine M. Ribic, David Holland, John Howell, Anthony Jevnikar, S. Joseph Kim, Greg Knoll, Brenda Lee, Jeffrey Zaltzman, Azim S. Gangji

**Affiliations:** 1 Division of Nephrology, Department of Medicine, McMaster University, Hamilton, Ontario, Canada; 2 Department of Medicine, School of Medicine, Queen’s University, Kingston, Ontario, Canada; 3 Astellas Pharma Canada, Inc, Markham, Ontario, Canada; 4 Division of Nephrology, Department of Medicine, Western University, London, Ontario, Canada; 5 Division of Nephrology, Department of Medicine, University of Toronto, Ontario, Canada; 6 Division of Nephrology, Department of Medicine, University of Ottawa, Ontario, Canada; 7 Clinical Epidemiology Program, Ottawa Health Research Institute, Ontario, Canada

**Keywords:** cardiovascular disease, mortality, renal transplant, risk factor

## Abstract

**Background:**

Renal transplant recipients (RTRs) are at significantly higher risk for morbidity and mortality compared with the general population, largely attributed to cardiovascular disease (CVD). Previous estimates of CVD events have come from health care databases and retrospective studies.

**Objective:**

The objective of this study was to prospectively determine the prevalence of risk factors and incidence of CVD events in a Canadian cohort of RTRs.

**Design:**

Study of Cardiovascular Outcomes in Renal Transplantation (SCORe) was a prospective, longitudinal, multicenter observational study.

**Setting:**

Adult RTRs were recruited from 6 participating transplant sites in Ontario, Canada.

**Patients:**

Eligible patients were those receiving a living or deceased donor renal transplant. Patients who received simultaneous transplant of any other organ were excluded.

**Measurements:**

Primary outcomes included myocardial infarction (MI) defined by American College of Cardiology (ACC-MI) criteria, and major adverse cardiac events (MACE), defined as cardiovascular (CV) death, ACC-MI, coronary revascularization, and nonhemorrhagic stroke. CV events were adjudicated by a single, independent cardiologist.

**Methods:**

CV and transplant-specific risk factors that predict MACE and ACC-MI were identified by stepwise regression analysis using the Cox proportional hazards model.

**Results:**

A total of 1303 patients enrolled across 6 transplant centers were followed for 4.5 ± 1.6 years (mean ± SD). Incidence of MACE was 7.0%, with significant independent predictors/risk factors including age, diabetes, coronary revascularization, nonhemorrhagic stroke, and renal replacement therapy (RRT). ACC-MI incidence was 4.0%, with significant independent predictors/risk factors including age, coronary revascularization, and duration of RRT in excess of the median value (2.91 years).

**Limitations:**

Patients were recruited from a single province, so may not reflect the experience of RTRs in other areas of Canada.

**Conclusions:**

Using a prospective design and rigorous methodology, this study found that the incidence of CV events after renal transplantation was elevated relative to the general Canadian population and was comparable with that reported in patient registries, thus helping validate the utility of retrospective analysis in this field. SCORe highlights the importance of monitoring RTRs for traditional cardiac and transplant-specific CV risk factors to help prevent CV morbidity and mortality. Further research is needed to investigate a broader range of potential risk factors and their combined effects on incident CV events.

## Abrégé

**Contexte:**

Les receveurs d’une greffe rénale présentent un risque significativement plus élevé de morbidité et de mortalité que l’ensemble de la population, ceci est en grande partie attribuable aux maladies cardiovasculaires. Les estimations antérieures du nombre d’événements cardiovasculaires proviennent des bases de données du système de santé et des résultats d’études rétrospectives.

**Objectif de l’étude:**

L’étude visait à mesurer de manière prospective la prévalence des facteurs de risque et l’incidence des événements cardiovasculaires dans une cohorte de patients receveurs d’une greffe rénale au Canada.

**Type d’étude:**

L’étude SCORe consistait en une étude observationnelle longitudinale menée de façon prospective au sein de plusieurs centres hospitaliers.

**Cadre de l’étude:**

Les patients adultes receveurs d’une greffe rénale ont été recrutés dans six hôpitaux de la province de l’Ontario, au Canada.

**Patients:**

Les patients qui avaient reçu une greffe rénale provenant d’un donneur vivant ou décédé ont été inclus dans l’étude. Les patients qui avaient reçu une greffe simultanée de tout autre organe ont été exclus.

**Mesures:**

Les principaux critères retenus incluaient un infarctus du myocarde répondant aux critères établis par l’American College of Cardiology (ACC-MI), des événements cardiaques majeurs indésirables (MACE) tels que le décès d’origine cardiovasculaire, l’ACC-MI, la revascularisation coronarienne et l’AVC non hémorragique. Ces événements cardiovasculaires ont été constatés par un cardiologue indépendant.

**Méthodologie:**

Les facteurs de risque, cardiovasculaires ou spécifiques à la transplantation, qui permettent de prédire les MACE et l’ACC-MI ont été identifiés à l’aide du modèle de risques proportionnels de Cox pour l’analyse de régression en étapes.

**Résultats:**

Les 1 303 patients recrutés au sein des six centres hospitaliers ont été suivis sur une moyenne de 1,6 an (SD ± 4,5). L’incidence de MACE était de 7,0%, avec des indicateurs prévisionnels indépendants et des facteurs de risque significatifs incluant l’âge, le diabète, la revascularisation coronarienne, l’AVC non hémorragique et la thérapie de remplacement rénale. L’incidence d’ACC-MI était de 4,0%, avec des indicateurs prévisionnels indépendants et des facteurs de risque significatifs incluant l’âge, la revascularisation coronarienne et une durée pour la thérapie de remplacement rénal excédant la valeur médiane (2,91 ans).

**Limites de l’étude:**

Les patients ont été recrutés dans une seule province, les résultats sont susceptibles de ne pas être représentatifs de la situation des receveurs d’une greffe rénale ailleurs au Canada.

**Conclusions:**

Grâce à un plan d’étude prospectif et à une méthodologie rigoureuse, nous avons déterminé que l’incidence des événements cardiovasculaires chez les receveurs d’une greffe rénale a été plus élevée que dans la population canadienne en général. Nous avons également constaté qu’elle était comparable à l’incidence rapportée dans les registres de patients, ce qui vient confirmer l’utilité des études rétrospectives dans ce domaine. Les résultats obtenus lors de l’étude SCORe mettent en lumière l’importance de faire un suivi auprès des receveurs d’une greffe rénale afin de détecter les facteurs de risque cardiovasculaires ou spécifiques à l’intervention, et ainsi aider à prévenir la mortalité et la morbidité liées aux maladies cardiovasculaires. D’autres recherches sont nécessaires afin d’examiner un plus large éventail de facteurs de risques potentiels et leurs effets concomitants sur les événements cardiovasculaires.

## What was known before

Based on retrospective and registry data, renal transplant recipients, compared with the general population, are at significantly higher risk for morbidity and mortality. These findings are largely attributed to cardiovascular (CV) events, which may be partially explained by traditional CV risk factors in this population and/or by other factors that are unique to renal transplantation or management in the posttransplantation setting.

## What this adds

Prospective data from Canadian renal transplant recipients in Ontario appear consistent with findings from other data sources. The study highlights the importance of identifying both baseline CV and transplant-specific risk factors for patients undergoing renal transplant, to help prevent CV events post transplant.

## Background

Estimates of cardiovascular disease (CVD) incidence in renal transplant recipients (RTRs) have mainly been derived from large registry databases or from retrospective studies.^1,2^ Despite pretransplant assessment and cardiac screening of this population, the incidence of CVD in RTRs is 3 to 5 times higher than in the age-matched general population.^2^ For patients treated with maintenance hemodialysis, the risk is 10 to 20 times higher than the general population,^2^ with the annual risk of death from CVD approximately 3.5% to 5% in RTRs.^3^ After transplantation, multiple cardiovascular (CV) risk factors prevalent among dialysis patients can persist and even increase.^3^

Retrospective and registry data have established that CVD, including ischemic heart disease and cerebrovascular disease (CBD), as well as peripheral vascular disease (PVD), causes significant morbidity and mortality in RTRs, but prospective longitudinal studies are still lacking. Evaluations of annual CVD rates from retrospective studies range from 0.4% in the United States to 3.0% in Scandinavia in RTRs.^4-6^ Limitations that may influence the interpretation of registry data include entry of unadjudicated diagnoses or multiple diagnoses, duplicate reports, reporting delays or missing data, and misclassification of patient characteristics.^7-9^

Study of Cardiovascular Outcomes in Renal Transplantation (SCORe) was a prospective, longitudinal, multicenter observational study designed to determine the incidence of CVD events in the RTR population in the general maintenance hemodialysis population in a single health care system (Ontario, Canada). The study also determined predictors of CVD events from both known CV risk factors and potential renal transplant–specific risk factors, and it compared observed findings with comparator populations.^10-12^

## Methods

### Patients and Data Collection

Adult patients were eligible for inclusion in SCORe if they received a living or deceased-donor renal transplant at any of 6 participating transplant sites in Ontario, Canada. Prior to participation in the trial, patients provided written informed consent, which could be completed before or after transplantation. Inclusion criteria were deliberately broad; patients who received simultaneous transplant of any other organ were excluded, but those who received prior renal transplants in the absence of other organ transplantations were considered eligible, and those participating in other therapeutic trials were not excluded. There were no exclusion criteria or restrictions placed on the immunosuppressive regimens.

Patients were evaluated at regular intervals as part of routine clinical care, starting at baseline (before or after transplantation), defined as the first study visit after consent, followed by 6 months post transplant, and then yearly thereafter, until either patient death, graft loss, or study completion, with data collected in a prospective manner. Patients who experienced graft failure (ie, permanent return to dialysis or nephrectomy) were followed for an additional 6 months for CVD outcomes. Those who experienced allograft failure and had a subsequent renal transplant were eligible for study reentry at the time of the subsequent transplant if all study eligibility criteria were met.

Baseline data collection occurred on the first study visit after consent. Information collected was related to the current study transplant, as well as the patient’s medical history. CVD outcome variables included chronic or unstable angina, myocardial infarction (MI), congestive heart failure (CHF), atrial fibrillation, coronary revascularization procedures, PVD, renal revascularization, CBD, nonhemorrhagic stroke, and death from CVD. All suspected MI events were adjudicated centrally by a single independent cardiologist, who reviewed all generated electrocardiograms (ECGs), echocardiograms, laboratory data, and admission notes at each follow-up time point. Potential CVD events were evaluated according to the American College of Cardiology (ACC) criteria^13^ and defined as ACC-MI if criteria were met. Deaths were adjudicated by 2 independent investigators using source data, and consensus was reached on discrepant classifications.

Recorded primary outcomes included ACC-MI and major adverse cardiac events (MACE), defined as CV death, nonfatal MI, ACC-MI, coronary revascularization, and nonhemorrhagic stroke.

### Statistical Analysis

The planned study population was 2000 de novo RTRs enrolled over a 5-year period starting from 2002. Demographic characteristics were collected at baseline and reported using descriptive statistics. Distributions were calculated for the baseline CV risk factors and transplant-specific characteristics. Continuous variables were reported using mean and standard deviation; categorical variables were reported using frequency and percentages. In supplementary sensitivity analyses, duration of renal replacement therapy (RRT), donor age, and time on the wait list were modeled as continuous variables. Incidence of MACE, ACC-MI, and each of the MACE components were calculated, as well as Kaplan-Meier estimates of the time to first qualifying MACE and ACC-MI.

Risk factors that predict MACE were determined by stepwise regression analysis using Cox proportional hazards modeling, carried out for a general model incorporating all baseline CV risk factors and transplant-specific factors. These baseline factors comprised age, sex, diabetes, hypertension, dyslipidemia, smoking history, MI, coronary revascularization, nonhemorrhagic stroke, PVD, and obesity (body mass index [BMI] >30 kg/m^2^). Transplant-specific factors included use of or duration of RRT, delayed graft function, time on wait list, immunosuppression protocol, graft rejection, donor kidney type (live or deceased), donor age, and prior renal transplant. Similarly, baseline CV risk factors and transplant-specific risk factors that predicted ACC-MI were determined by stepwise regression analysis using Cox proportional hazards modeling, carried out for the general model, as above.

Independent predictors of MACE were identified by stepwise regression analysis in a reduced model incorporating any factors in the general model that satisfied *P* ≤ .05 for association with incident MACE. Independent predictors of ACC-MI were identified in a reduced model incorporating factors that satisfied *P* ≤ .05 for association with incident ACC-MI.

In supplementary sensitivity analyses, the same set of baseline CV risk factors were analyzed for MACE and ACC-MI, using variant models, such as treating unknown obesity status as missing or including time on wait list, donor age, and duration of RRT as continuous, rather than categorical, variables.

### Comparator Populations

The predefined analysis plan included comparison of baseline characteristics and CV outcomes with reference populations, including high-risk patients from the international Heart Outcomes Prevention Evaluation (HOPE) trial (an interventional study testing the cardioprotective effect of the angiotensin-converting enzyme inhibitor ramipril)^10^ and the general Canadian population.^12^ Subsequently, findings from the Canadian Organ Replacement Register (CORR) cohort were published,^11^ so the comparison was extended to include this reference population.

## Results

### Study Population

Enrollment of consecutive patients undergoing renal transplantation at 6 transplant centers in Ontario, Canada, began in September 2003, with 27 patients enrolled until the end of the year. From January 1, 2004, to October 31, 2008, the enrollment rate varied between 202 and 299 patients/y. A total of 1301 individuals, comprising 1303 distinct kidney transplantation cases (2 patients underwent retransplantation during the study period), were included in this study. Per-site enrollment ranged from 32 (2.5% of the total study population) to 354 (27.2%) cases. Mean follow-up time was approximately 4.5 years. Overall, a total of 1224 (93.9%) patients were followed-up up to 2 years, 1082 (83.0%) up to 3 years, 772 (59.2%) up to 4 years, and 511 (39.2%) up to 5 years or to the end of the study.

Baseline demographics are summarized in Table 1. The mean ± SD age at the time of transplant was 49.3 ± 13.2 years, and the most common cause of end-stage renal disease (ESRD) was reported as glomerulonephritis. Approximately half of the patients received hemodialysis only. Most RTRs experienced hemodialysis and/or peritoneal dialysis before kidney transplantation, with a mean ± SD duration of RRT 3.7 ± 3.4 years and median time on dialysis of 2.9 years; a minority of RTRs (13.6%) received preemptive transplantation. Mean ± SD time on the wait list for recipients in this study was 3.1 ± 2.9 years.

**Table 1.** Baseline Demographics.

Characteristic	SCORe (N = 1303)
Recipient age, y, mean (SD)	49.3 (13.22)
Sex, male, n (%)	838 (64.3)
Race, n (%)	
Caucasian	927 (71.1)
Asian	103 (7.9)
African	98 (7.5)
Mixed, Unknown, or Other	91 (7.0)
East Indian	66 (5.1)
North American Aboriginal	18 (1.4)
Cause of ESRD, n (%)	
Glomerulonephritis	545 (41.8)
Diabetic nephropathy	214 (16.4)
PCKD	170 (13.1)
Reflux nephropathy	113 (8.7)
Hypertension	100 (7.7)
Drug induced	16 (1.2)
Other	145 (11.1)
Dialysis modality, n (%)	
Hemodialysis only	718 (55.1)
Peritoneal dialysis only	232 (17.8)
Preemptive transplant	177 (13.6)
Hemodialysis and peritoneal dialysis	176 (13.5)
Time on dialysis, y, mean (SD); median (range)	3.7 (3.4); 2.9 (0.0-31.6)
Age of donor, y, mean (SD; median (range)	44.6 (13.2); 46.0 (3-80)
Time on wait list, y, mean (SD); median (range)	3.1 (2.9); 2.1 (0.0-18.5)

*Note.* SCORe = Study of Cardiovascular Outcomes in Renal Transplantation; ESRD = end-stage renal disease; PCKD = polycystic chronic kidney disease.

Table 2 summarizes baseline distribution of traditional CV risk factors and potential renal transplant–specific risk factors. Most RTRs (64.3%) were male, and approximately one-half had known CV risk factors (eg, age ≥50 years, hypertension, or a history of smoking). The mean donor age was 44.6 years (±13.2), and in 48.5% of renal transplants, the source was a living donor. The mean ± SD serum creatinine values at weeks 1 and 2 post transplant were 215.8 ± 192.8 µmol/L and 168.5 ± 122.6 µmol/L, respectively (data not shown). Most patients received induction therapy in the form of lymphocyte-depleting agents or interleukin 2 (IL-2) receptor blockers.

**Table 2.** Distribution of Baseline Risk Factors for CVD.

Risk factor	Number (%)	95% CI
Known CV risk factors		
Age (≥50 y)	678 (52.0)	49.3%-54.8%
Sex (% male)	838 (64.3)	61.6%-66.9%
Hypertension (SBP ≥140 or DBP ≥90 mmHg)	649 (49.8)	47.1%-52.6%
Diabetes (currently receiving treatment)	284 (21.8)	19.6%-24.2%
Dyslipidemia (currently receiving antilipid therapy)	523 (40.1)	37.6%-43.1%
Smoking (ever)	662 (50.8)	48.1%-53.6%
Previous CV event (includes angina, MI)	139 (10.7)	9.6%-13.2%
MI	83 (6.4)	5.1%-7.8%
Cerebrovascular accident	30 (2.3)	1.6%-3.3%
Nonhemorrhagic stroke	23 (1.8)	1.1%-2.6%
Coronary revascularization	119 (9.1)	7.6%-10.8%
Peripheral vascular disease	31 (2.4)	1.6%-3.4%
Endarterectomy	4 (0.3)	0.1%-0.8%
Obesity (BMI >30 kg/m^2^)	317 (24.3)	22.5%-27.3%
Potential renal transplant–specific risk factors		
Duration of renal replacement therapy (≥median)	606 (46.5)	47.2%-52.9%
Source of kidney (living vs deceased)	632 (48.5)	45.8%-51.3%
Donor age (≥median)	511 (39.2)	47.6%-53.8%
Prior renal transplant	143 (11.0)	9.3%-12.8%
Delayed graft function	195 (15.0)	13.5%-17.6%
Induction therapy within first 2 wk		
ATG, OKT3	373 (28.6)	26.2%-31.2%
Basiliximab, daclizumab	473 (36.3)	33.7%-39.0%
Immunosuppressive therapy in first 2 wk		
CNI: cyclosporine/tacrolimus	1244 (95.5)	94.2%-96.5%
Antimetabolite: azathioprine, MMF	1254 (96.2)	95.1%-97.2%
MTOR inhibitor: sirolimus	32 (2.5)	1.7%-3.5%
Steroids	1261 (96.8)	95.7%-97.7%
Time on wait list (≥median)	601 (46.1)	47.2%-53.0%
Graft rejection	230 (17.6)	15.6%-19.8%

*Note.* CVD = cardiovascular disease; CI = confidence interval; CV = cardiovascular; SBP = systolic blood pressure; DBP = diastolic blood pressure; BMI = body mass index; MI = myocardial infarction; ATG = antithymocyte globulin therapy; OKT3 = murine monoclonal antibody of the immunoglobulin IgG2a isotype; CNI = calcineurin inhibitor; MMF = mycophenolate mofetil; MTOR = mammalian target of rapamycin.

### Outcomes

**Figure 1.**
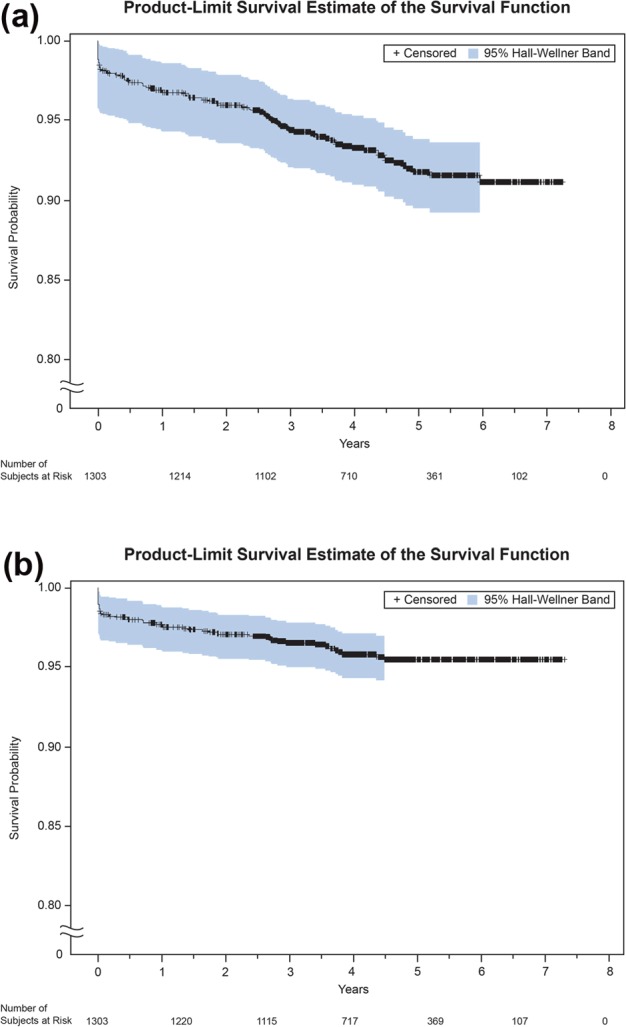
Kaplan-Meier curves of time to CV event. *Note.* (a) Time to first MACE event and (b) time to first ACC-MI. Number of patients at risk was assessed at 0, 500, 1000, 1500, 2000, 2500, and 3000 days. CV = cardiovascular; MACE = major adverse cardiac events; ACC = American College of Cardiology.

During the observation period, 91 (7.0%) patients experienced a total of 137 MACE events, including 52 (4%) who experienced 63 ACC-MI events (Table 3). The incidence of MACE was 23.4 per 1000 person-years, whereas the incidence of ACC-MI was 10.8 per 1000 person-years. Figure 1 shows the Kaplan-Meier curves of time to first MACE and ACC-MI events, indicating an excess of events immediately following transplantation. Indeed, 23 of the 91 MACE events and 21 of the 52 ACC-MI events occurred within the first 2 weeks following renal transplantation; events continued to occur at a reduced rate thereafter. There were 24 (1.8%) deaths from CVD among 86 RTRs (6.6%) who died during the follow-up period. Of these, 1 was adjudicated as the result of an ACC-MI. Due to the observational nature of the study, other fatal CV events may have lacked the necessary assessments to determine whether they met ACC criteria.

**Table 3.** Number and Incidence of MACE and ACC-MI Events Among Renal Transplant Recipients.

Event	Incidence of individual and composite events, n (%)	Total events^a^	Incidence per 1000 person-years
MACE	91 (7.0)	137	23.4
CV death	24 (1.8)	24	4.1
Nonfatal ACC-MI	51 (3.9)	62	10.6
Coronary revascularization	37 (2.8)	42	7.2
Nonhemorrhagic stroke	8 (0.6)	9	1.5
ACC-MI	52 (4.0)	63	10.8

*Note.* MACE = major adverse cardiac events; ACC = American College of Cardiology; MI = myocardial infarction; CV = cardiovascular.

a Includes patients who experienced more than 1 event.

Other deaths were due to malignancy (22, 1.7%), infection (11, 0.8%), hemorrhagic stroke (3, 0.2%), multiorgan failure (2, 0.2%), respiratory failure (2, 0.2%), and bleeding (1, 0.08%), with 6 (0.5%) deaths due to other causes and 15 (1.1%) to unknown causes.

### Predictors of MACE and ACC-MI

A stepwise regression analysis of MACE identified 4 independent predictors from known CV risk factors: age, diabetes, coronary revascularization, and nonhemorrhagic stroke. A similar stepwise regression analysis of ACC-MI identified age and coronary revascularization as independent baseline risk factors. Patient age was strongly correlated with both outcomes (*P* < .0001 for MACE and *P* = .029 for ACC-MI).

To expand the risk models for MACE and ACC-MI beyond known CV risk factors, potential renal transplant–specific factors were included with the baseline predictors of MACE and ACC-MI. For both outcomes, RRT (modeled as a categorical event for MACE or as a dichotomized, time-dependent factor for ACC-MI) emerged as a significant and independent CV risk factor (Table 4).

**Table 4.** Independent Predictors of MACE and ACC-MI.

Risk factor	Hazard ratio		Incidence (% of patients in study population)	*P* value
	Point estimate	95% CI		
MACE				
Age, y	1.04	1.02-1.06	—	<.0001
Diabetes (yes vs no)	1.9	1.2-2.8	21.8	.004
Coronary revascularization (yes vs no)	2.4	1.4-3.8	9.1	.0005
Nonhemorrhagic stroke (yes vs no)	3.5	1.4-7.3	1.8	.002
RRT (dialysis vs preemptive transplant)	8.1	2.2-71.0	86.4	.012
ACC-MI				
Age, y	1.03	1.003-1.05	—	.029
Coronary revascularization (yes vs no)	3.2	1.7-5.9	9.1	.0002
Duration of RRT (≥median vs <median)	3.2	1.6-6.6	46.5	.001

*Note.* MACE = major adverse cardiac events; ACC = American College of Cardiology; MI = myocardial infarction; RRT = renal replacement therapy.

In supplementary analyses where duration inputs were modeled as continuous, rather than dichotomous, variables, the independent risk factors for MACE and for ACC-MI were generally similar to those shown in Table 4. However, for MACE, nonhemorrhagic stroke was excluded as an independent risk factor in the sensitivity analysis, and duration of RRT (but not use of RRT) was included. For ACC-MI, age was excluded as an independent risk factor in this sensitivity analysis. An additional sensitivity analysis, in which missing obesity status was coded as unknown, had no effect on the models of MACE or ACC-MI.

### Comparison of SCORe and Reference Populations

As shown in Table 5, traditional CV risk factors such as hypertension, diabetes, and obesity were more prevalent among SCORe patients than in the general Canadian population, whereas some risk factors (prior MI and PVD) were markedly more common in the HOPE study patients than in SCORe. Patients registered in CORR, representing another Canadian renal transplant data set from overlapping years, showed CV risk factor prevalences that were generally similar to those in SCORe. Patients in the CORR data set had a lower incidence of MI and CBD, relative to SCORe patients. Otherwise, the incidence of CV events in the SCORe population followed a similar pattern to that of the risk factor prevalence in the CORR database. Thus, where comparisons were possible, each event type (CV death, total and nonfatal MI, and CBD) occurred more frequently than in the general Canadian population but less frequently than was reported for the HOPE study patients.

**Table 5.** Prevalence of CV Risk Factors and Incidence of CVD Events Observed Prospectively in SCORe Compared With Reference Populations.

Risk factor	SCORe	CORR (2002-2005)^11,a^	CORR (2006-2009)^11,a^	HOPE^10,b^	Canadian population^12,c^
Prevalence of risk factors, %					
MI	6.4	2.7	2.3	52.6	—
CBD^d^	2.3	1.2^e^	1.2^e^	10.9	—
Hypertension	49.8	74	69	46.8	14.6
Diabetes	21.8	27	29	38.5	3.6
Smoking	50.8	—	—	14.2	18.2
Peripheral vascular disease	2.4	—	—	43.6	—
Obesity (BMI ≥30 kg/m^2^)	24.3	—	—	—	11.2
Incidence of CVD events per 1000 person-years					
CVD death	4.1			18.3	2.5
Nonfatal MI	8.7				2.1
MI	8.9	1.4	1.5	28.6	
CBD^d^	1.7	0.9^f^	0.5^f^	10.6	

*Note.* CV = cardiovascular; SCORe = Study of Cardiovascular Outcomes in Renal Transplantation; CORR = Canadian Organ Replacement Register; HOPE = Heart Outcomes Prevention Evaluation; MI = myocardial infarction; CBD = cerebrovascular disease; BMI = body mass index.

a Incidence over 4-year period indicated.

b Incidence based on approximate study period, 4.5 years.

c Incidence in year 2004.

d Cerebrovascular disease due to ischemic or hemorrhagic stroke, except as indicated.

e Stroke or transient ischemic attack.

f Ischemic stroke.

## Discussion

To date, information on CV outcomes in RTRs has been limited to retrospective analyses, but uniquely, SCORe prospectively collected data from more than 1300 consecutive patients at all Ontario centers, followed for a mean of 4.5 years. SCORe participation was open to patients irrespective of prior transplant history or prior or concurrent participation in any other study. Despite the rigorous methodology used in SCORe, which included adjudication by a single cardiologist, the study found CV event incidence was not increased, relative to retrospective data from comparator populations. This finding provides significant reassurance about the validity of retrospective outcome data; specifically, we find no support for the long-standing suspicion that published Canadian renal transplant registry data might underreport the true incidence of CV events.

Prespecified comparisons of risk factor prevalence and CV event incidence were carried out between SCORe and the general Canadian population (representing baseline CV risk) and between SCORe and the patient population from the HOPE study,^10^ which followed >9000 individuals deemed at inclusion (1993-1995) to be at high risk of CV events. As expected, CV incidence in SCORe appeared intermediate between the low- and high-risk comparator populations. In addition, with the publication of the CORR database analysis in 2016,^11^ it was possible to directly compare CV event incidence in retrospectively identified Ontario renal transplant patients with the prospectively ascertained SCORe population. Notably, CORR findings were divided by 4-year intervals, and 2 of these intervals overlapped with the observation period for SCORE. Comparison of SCORe and CORR data showed substantially greater prevalence of prior MI and likewise a higher incidence of MIs in the SCORE population.

The highest rate of CVD events occurred in the immediate (2-week) post-transplant period. Most ACC-MI were nonfatal, and the majority of deaths were not cardiac in nature. Mortality secondary to CVD accounted for 27.9% (n = 24) of the 86 deaths, corresponding to a rate of 4.1 per 1000 person-years. This is slightly lower than registry data, which suggest that CVD accounts for up to 36% of deaths with a functioning graft.^14^ In Canada, patients with a functioning allograft had an unadjusted mortality rate of 2.06 per 100 patient-years, but this increased to 5.14 per 100 patient-years with patients with allograft failure.^15^ In comparison, for individuals with functioning grafts, the United States Renal Data System reported all-cause mortality at a rate of 31.9 per 1000 patient-years at risk in 2011.^16^ Perhaps, the low incidence of myocardial infarcts and CVD death seen postoperatively in our population can be attributed to the cardiac screening strategies employed before transplantation.

Known risk factors for CVD that SCORe identified as predictors of MACE and ACC-MI were similar to those previously reported in the literature.^3,17-19^ SCORe showed patient age was strongly correlated with both MACE and ACC-MI, such that for every 1-year increase in age, the hazard of MACE and ACC-MI increased by 4% and 3%, respectively. SCORe identified the renal transplant–specific factor of RRT as a predictor for both MACE and ACC-MI. These findings are similar to retrospective studies, such as that from the Netherlands, which found independent risk factors for posttransplant CV events included diabetic nephropathy, claudication, time-on-dialysis, recipient age, and BMI.^18^

SCORe findings are also consistent with data from a Spanish study^20^ showing that age, smoking, diabetes, and postsurgical left ventricular (LV) hypertrophy were independent predictors of CV events in RTRs. Prolonged duration of dialysis has consistently been associated with worse CVD outcomes,^17-19^ and this was replicated in our study. Treatment for hyperlipidemia was not significantly associated with a reduction in MACE or ACC-MI in our cohort, nor in previously published literature.^21^ This may reflect the contribution of nonatherosclerotic mechanisms in the pathogenesis of MACE and ACC-MI events.

The strengths of SCORe include the prospective collection of CVD outcomes data from more than 1300 consecutively screened kidney transplant patients. In addition, all patients were recruited from the province of Ontario, which provides uniform health care access. Unique to this study, all cases in the large data set were adjudicated by a single cardiologist, and there were no secondary data sources used, nor imputation of missing data. A clinically relevant CV end point, ACC-MI, was used. Multiple sensitivity analyses were carried out in the hazard analysis, with generally good agreement related to independent risk factors for ACC-MI and MACE.

Study limitations included a limited power to detect a significant effect of some potentially important CV risk factors. Comparator data, notably from the HOPE study,^10^ were collected at an earlier time, relative to the observation period for SCORe. Conversely, for the CORR study data,^11^ the intervals selected for comparison overlapped with timing of SCORe. Statistical significance was not analyzed for the observed differences among these divergent comparator populations. Further efforts to define the CV risk profile of renal transplant patients should look beyond the factors considered in our analysis and focus on other potential risk factors that have emerged from recent association studies.^22,23^ Finally, as all patients were recruited from the province of Ontario, our findings many not fully reflect the experience in other areas of Canada or in other health care settings.

## Conclusions

In conclusion, as a large, prospective study, SCORe adds to and provides support for an evidence-based body of literature that is otherwise largely derived from retrospective analyses. SCORe data confirm that CVD seen postoperatively after renal transplantation is predicted by pretransplantation factors including age, coronary revascularization, and dialysis use, and they highlight the importance of effective and comprehensive pretransplantation screening strategies.
